# Cognitive-affective network structure in adolescents with non-suicidal self-injury: implications for clinical intervention

**DOI:** 10.3389/fpsyt.2026.1804570

**Published:** 2026-06-30

**Authors:** Shen Li, Lijun Wang, Wenjie Sun, Yuxin Han, Nannan Liu, Xinxu Wang, Jie Li, Xiangyang Zhang

**Affiliations:** 1 Institute of Mental Health, Tianjin Anding Hospital, Mental Health Center of Tianjin University, Tianjin, China; 2 Brain Assessment & Intervention Laboratory, Tianjin Anding Hospital, Mental Health Center of Tianjin University, Tianjin, China; 3 Institute of Mental Health, Tianjin Anding Hospital, Mental Health Center of Tianjin Medical University, Tianjin, China; 4 Hefei Fourth People’s Hospital, Anhui Mental Health Center, Affiliated Mental Health Center of Anhui Medical University, Hefei, Anhui, China

**Keywords:** clinical intervention, cognitive fusion, experiential avoidance, network analysis, non-suicidal self-injury

## Abstract

**Background:**

Non-suicidal self-injury (NSSI) among adolescents represents a pressing global mental health challenge. Despite its high prevalence, the cognitive-affective mechanisms underlying NSSI remain poorly understood. Experiential avoidance (EA) and cognitive fusion (CF) represent key cognitive-affective processes implicated in NSSI vulnerability. This study examined the network architecture linking cognitive and affective components of EA and CF in adolescents with NSSI, to identify central and bridging elements that may inform clinical intervention strategies.

**Methods:**

A total of 4023 adolescents were recruited through a large-scale epidemiological survey. Network analysis was conducted within the subgroup of 1387 adolescents reporting NSSI in the past 12 months. NSSI behaviors were assessed using the Adolescent Non-Suicidal Self-Injury Assessment Questionnaire. EA and CF were measured using the Acceptance and Action Questionnaire-II and the Chinese version of the Cognitive Fusion Questionnaire, respectively. Network analysis was conducted to map the cognitive-affective network structure and identify central nodes (most interconnected components) and bridge nodes (linking cognitive and affective clusters).

**Results:**

Within the NSSI subgroup, the cognitive-affective network revealed that thought distress [Expected Influence (EI) = 1.13] emerged as the most central node, showing strong connections to inner struggle (weight = 0.25), conflicting thoughts (weight = 0.22), and painful thoughts (weight = 0.22). Bridge analysis identified painful thoughts [Bridge Expected Influence (BEI) = 0.44], emotional interference (BEI = 0.43), rumination and procrastination (BEI = 0.40), and task disruption (BEI = 0.39) as key bridging nodes linking cognitive and affective processes. The EI stability coefficient was 0.75, indicating good reliability. Network structure was invariant across sex.

**Conclusion:**

Thought distress occupies a central position in the cognitive-affective network of adolescent NSSI, while painful thoughts, emotional interference, rumination, and task disruption serve as key bridges between cognitive and affective domains, with bridging nodes spanning both EA and CF communities. These network-based findings highlight potential intervention targets that may help disrupt maladaptive cognitive-affective patterns in adolescents who engage in NSSI.

## 1 Introduction

Non-suicidal self-injury (NSSI), the deliberate infliction of harm on one’s body without suicidal intent, is a pervasive and pressing mental health issue among adolescents. Behaviors such as cutting, burning, hitting, scratching, or hair pulling are not only highly distressing but also associated with elevated risks of psychiatric comorbidities and future suicidal behaviors (1). Recognizing its clinical importance, the DSM-5 included NSSI as a condition for further study, reflecting growing consensus on its distinct psychopathological profile (2). Although sociocultural influences contribute to its development, recent large-scale studies engaging in NSSI (3). Despite its widespread impact, the underlying psychological mechanisms that sustain NSSI remain poorly understood.

Emerging evidence points to psychological inflexibility, particularly EA and CF, as key processes involved in NSSI behaviors (4, 5). EA is defined as an individual’s tendency to escape, suppress, or control negative internal experiences, such as distress, anxiety, or fear (6). While avoidance may offer short-term relief, it often contributed to increased psychological distress over time and plays a central role in various psychiatric disorders, including depression and anxiety (7). CF, in contrast, refers to the tendency to become excessively entangled with one’s thoughts, perceiving them as literal truths rather than transient mental events (8). While both constructs have been implicated in emotional dysregulation and self-injurious behaviors, prior studies have predominantly relied on total scores or latent variable models, obscuring the nuanced interplay between specific cognitive-affective components (4, 8, 9). Moreover, the item-level associations between EA and CF in NSSI populations remain underexplored.

Recent advancements in network analysis have introduced a data-driven psychometric approach to explore complex relationships among psychological variables. In psychometric network analysis, psychological symptoms or constructs are represented as nodes, while the connections (edges) between them indicate partial correlations (10–12). Unlike traditional latent variable models, network analysis allows for the identification or central and bridge symptoms that may play pivotal roles in psychopathology. Applying this framework to NSSI offers an opportunity to move beyond aggregate measures and to reveal fine-grained psychological mechanisms that sustain self-injurious behavior.

To the best of our knowledge, few previous studies have comprehensively examined the item-level relationship between EA and CF in adolescents with NSSI. Specifically, we aim to identify the most central and bridge nodes within the EA-CF network, thereby providing a more refined understanding of the psychological mechanisms underlying NSSI.

## 2 Methods

### 2.1 Participants

The cross-sectional study utilized data from a large-scale epidemiological survey conducted in China. A total of 4, 043 adolescent participants completed online questionnaire, with only 20 responses excluded due to missing data. Therefore, the final sample comprised 4, 023 adolescents (mean age 16.1 ± 0.9 years, with 49.1% males). Inclusion criteria: 1. A secondary vocational school student; 2. voluntary and cooperation; Exclusion Criteria: 1. a history of mental illness; 2. previous suicidal behavior. These exclusions were applied *a priori* to characterize NSSI in its community, non-clinical form and to reduce confounding from psychotropic medication or active suicidality. All criteria were evaluated using self-reported methods. Participants were categorized into two groups based on their self-reported NSSI behaviors, assessed using the Adolescent Non-Suicidal Self-Injury Assessment Questionnaire (ANSAQ) scale. Individuals reporting at least one instance of self-injury were classified into the NSSI group; those reporting no such behavior were placed in the control group.

The study protocol was approved by the Ethics Committee of Tianjin Anding Hospital (Approved ID: 2024-02). Written informed consent was obtained from all participants, in accordance with the ethical principles of the Declaration of Helsinki.

### 2.2 Measures

#### 2.2.1 The assessment of NSSI

NSSI was assessed using the ANSAQ (13), which comprises two sections: the Behavior Questionnaire and the Function Questionnaire. The Behavior Questionnaire consists of 12 items assessing the frequency of specific self-injurious behaviors. It has demonstrated strong psychometric properties, including a Cronbach’s α coefficient of 0.92, split-half reliability coefficient of 0.85, and test-retest reliability coefficient of 0.84. The cumulative variance contribution rate was 64.91%, and the behavior score showed a significant correlation with the Functional Assessment of Self-Mutilation (FASM) behavior score (r=0.83, *p* < 0.01). A threshold of at least one occurrence within the past 12 months was used to determine the presence of NSSI. The Function Questionnaire comprises 19 items assessing the underlying functions of self-injury. It demonstrated strong internal consistency (Cronbach’s α coefficient of 0.91), split-half reliability coefficient of 0.79, and test-retest reliability coefficient of 0.81. The cumulative variance contribution rate was 53.9%, and the function score correlated strongly with the FASM behavior score (r = 0.86, *p* < 0.01). Responses are recorded on a five-point Likert scale ranging from 1 (none) to 5 (always), with higher scores indicating more severe NSSI behavior. In the current study, the Cronbach’s α coefficients were 0.91 for the Behavior Questionnaire and 0.86 for the Function Questionnaire, supporting the ANSAQ’s reliability and validity as an assessment tool for NSSI and its functions among Chinese adolescents.

#### 2.2.2 The assessment of experiential avoidance

Experiential avoidance was assessed using the Chinese version of the Acceptance and Action Questionnaire-II (AAQ-II), adapted by 14. based on the original instrument developed by Bond et al. (15).This 7-item scale uses a seven-point Likert response format, with higher scores reflecting greater EA. The AAQ-II has shown robust psychometric properties, including a Cronbach’s α of 0.88, test-retest reliability of 0.80, and explained variance of 62.5%. Concurrent validity has been supported by significant positive correlations with the Self-Rating Depression Scale (SDS; r=0.56, *p* < 0.01) and the Self-Rating Anxiety Scale (SAS; r = 0.55, *p* < 0.01). In the present study, the AAQ-II demonstrated excellent internal consistency (Cronbach’s α =0.93).

#### 2.2.3 The assessment of cognitive fusion

Cognitive fusion was measured using the Chinese version of the Cognitive Fusion Questionnaire (16), adapted from the original instrument developed by 17. The CFQ consists of 9 items rated on a 7-point Likert scale, with higher scores indicating greater CF. The scale has demonstrated strong psychometric characteristics, including a Cronbach’s α of 0.92, test-retest reliability of 0.67, and cumulative variance explanation of 60.3%. The CFQ has shown good concurrent validity, correlating positively with SDS (r = 0.50, *p* < 0.01) and SAS scores (r=0.55, *p* < 0.01). In the current study, the CFQ demonstrated excellent internal consistency (Cronbach’s α =0.96).

### 2.3 Statistical analysis

All network analyses were estimated within the NSSI subgroup (N = 1, 387). The present study aims to characterize the internal cognitive-affective architecture among adolescents who already engage in NSSI, for whom EA/CF-targeted interventions are clinically indicated. Estimating a pooled network across NSSI and non-NSSI participants would confound between-group differences with within-group symptom interrelations, obscuring the structure of interest.

Network analysis was conducted using the R package *qgraph* (version 1.9.8) (12, 18). In the analysis, nodes represent individual items from the AAQ-II and CFQ scales, and edges denoted partial correlations between nodes while controlling for all other associations. The strength of these correlations was visualized through the thickness of the edges, with thicker edges indicating stronger associations. Multivariate correlations were computed, and the network structure was estimated using the Gaussian graphical model (19). Node placement was determined via the Fruchterman-Reingold algorithm, which optimizes layout based on edge weights (20). To reduce the likelihood of spurious edges and improve interpretability, we applied the Least Absolute Shrinkage and Selection Operator (LASSO) regularization (21, 22) combined with the Extended Bayesian Information Criterion (EBIC) for model selection (23, 24). This procedure shrinks weaker edge weights to zero, yielding a sparse and stable network (22). Among 100 candidate models with varying sparsity, the model with the lowest EBIC value was selected (11). All network estimations were based on Spearman’s correlation coefficients.

Node centrality was assessed using the Expected Influence (EI) metric computed with the *qgraph* package. Unlike traditional centrality indices (e.g., strength, closeness, betweenness centrality), EI accounts for both positive and negative edge weights, offering a more robust measure of node influence within a psychological network (25, 26). EI reflects the sum of all edge weights connected to a node (27), with higher values indicating greater overall influence (26).

To identify bridging symptoms between EA and CF, we calculated Bridge Expected Influence (BEI) and Bridge strength using the R package *networktools* (version 1.5.2). BEI quantifies the extent to which a node in one community (e.g., EA) influences nodes in another community (e.g., CF), based on one-step connections (28). To minimize confirmation bias, we applied an 80^th^ percentile blind cutoff for the one-step BEI values for the identification of bridging nodes (29).

The accuracy and stability of the estimated networks were evaluated using nonparametric bootstrapping via the *bootnet* package (version 1.6) (12, 18). We computed 95% confidence intervals (CI) for edge weights and asses the stability of EI metrics using the centrality stability (CS) coefficient. A CS coefficient above 0.25 is considered acceptable, while values greater than 0.5 indicate excellent stability (18). To examine potential sex differences in network structure, we performed a Network Comparison Test (NCT) using the *NCT* package (version 2.2.2). This involved 3 sub-tests: 1) the network structure invariance test, assessing the largest absolute difference in edge strength within the network; 2) the global strength invariance test, comparing the overall connectivity; and 3) the edge strength invariance test, evaluating differences in specific edges weights (30). For the latter, the false discovery rate (FDR) correction was applied to adjust for multiple comparisons.

Additionally, we performed independent samples t-tests to compare scores between male and female participants.

To examine whether the network structure was confounded by comorbid depression and anxiety, we conducted a sensitivity analysis. Specifically, we regressed Patient Health Questionnaire-9 (PHQ-9) and Generalized Anxiety Disorder-7 (GAD-7) total scores from each EA and CF item using ordinary least squares regression and re-estimated the EBICglasso network using the residualized scores. We then compared the original and adjusted networks by examining Spearman rank-order correlations for node-level EI and BEI values, as well as the overall edge weight correlation between the two networks.

## 3 Results

### 3.1 Participants characteristics

This cross-sectional study included 4023 adolescents, of whom 1387 adolescents had engaged in NSSI behaviors in the past year. Participants’ levels of EA and CF were measured using scales, and a network psychometric approach was employed to estimate the regularized correlation network. **Table 1** presents a comparison of the epidemiological characteristics between the self-injury and non-self-injury groups, while **Table 2** lists the sex-based epidemiological characteristics of the 1387 adolescents with a history of self-injury. **Supplementary Table 1** shows the descriptive statistics for all analyzed variables.

**Table 1:** Demographic characteristics of the samples.

Variables	NSSI_Yes (N = 1387)	NSSI_No (N = 2636)
Age	16.01 ± 0.91	16.08 ± 0.91
Sex		
Male	667	1308
Female	720	1328
Experiential Avoidance		
Below cut-off (AAQ-ii < 25)	689	2099
Above cut-off (AAQ-ii ≥ 25)	698	537
Cognitive Fusion (CFQ)	36.17 ± 11.5	27.36 ± 10.49
Depressive Symptoms (PHQ-9)		
Below cut-off (PHQ-9 < 10)		
Absent	14	301
Minimal	200	1005
Mild	535	956
Above cut-off (PHQ-9 ≥ 10)		
Moderate	371	295
Moderate-to-severe	189	69
Severe	78	10
Anxiety Symptoms (GAD-7)		
Below cut-off (GAD-7 < 10)		
Absent	74	705
Minimal	380	1134
Mild	577	639
Above cut-off (GAD-7 ≥ 10)		
Moderate	255	130
Severe	101	28

N = 4023, GAD-7, Generalized Anxiety Disorder-7; PHQ-9, Patient Health Questionnaire-9; AAQ-ii, Acceptance and Action Questionnaire-version 2(AAQ-ii); CFQ, Cognitive Fusion Questionnaire(CFQ).

**Table 2:** Sex difference characteristics of the samples.

Variables	Male(N = 667)	Female(N = 720)
Age	16.1(0.94)	15.9(0.91)
PHQ-9	8.6(5.2)	10.9(5.4)
GAD-7	6(4.3)	7.87(4.9)
AAQ-ii	23.8(8.4)	27.4(8.2)
CFQ	33.6(11.3)	38.51(11.2)
NSSI frequency		
Non-obvious	3.99(4.0)	5.11(5.2)
Obvious	0.97(2.3)	1.96(2.9)
NSSI function		
Self-serving socializing	13.49(8.3)	15.2(7.4)
Self-negative Reinforcement	6.25(4.3)	6.82(3.9)
Emotional Expression	5.36(4.0)	6.11(3.9)

Values in parentheses indicate standard deviations; GAD-7, Generalized Anxiety Disorder -7; PHQ-9, Patient Health Questionnaire -9; AAQ-ii, Acceptance and Action Questionnaire – version 2 (AAQ-ii); CFQ, Cognitive Fusion Questionnaire (CFQ).

Sex differences were observed among participants with a history of self- injury across all variables. Significant sex differences were found in EA and CF for each subscale, with females demonstrating significantly higher scores than males (*p* < 0.001) (See **Supplementary Table 2**).

### 3.2 Network structure overview

**Figure 1:**
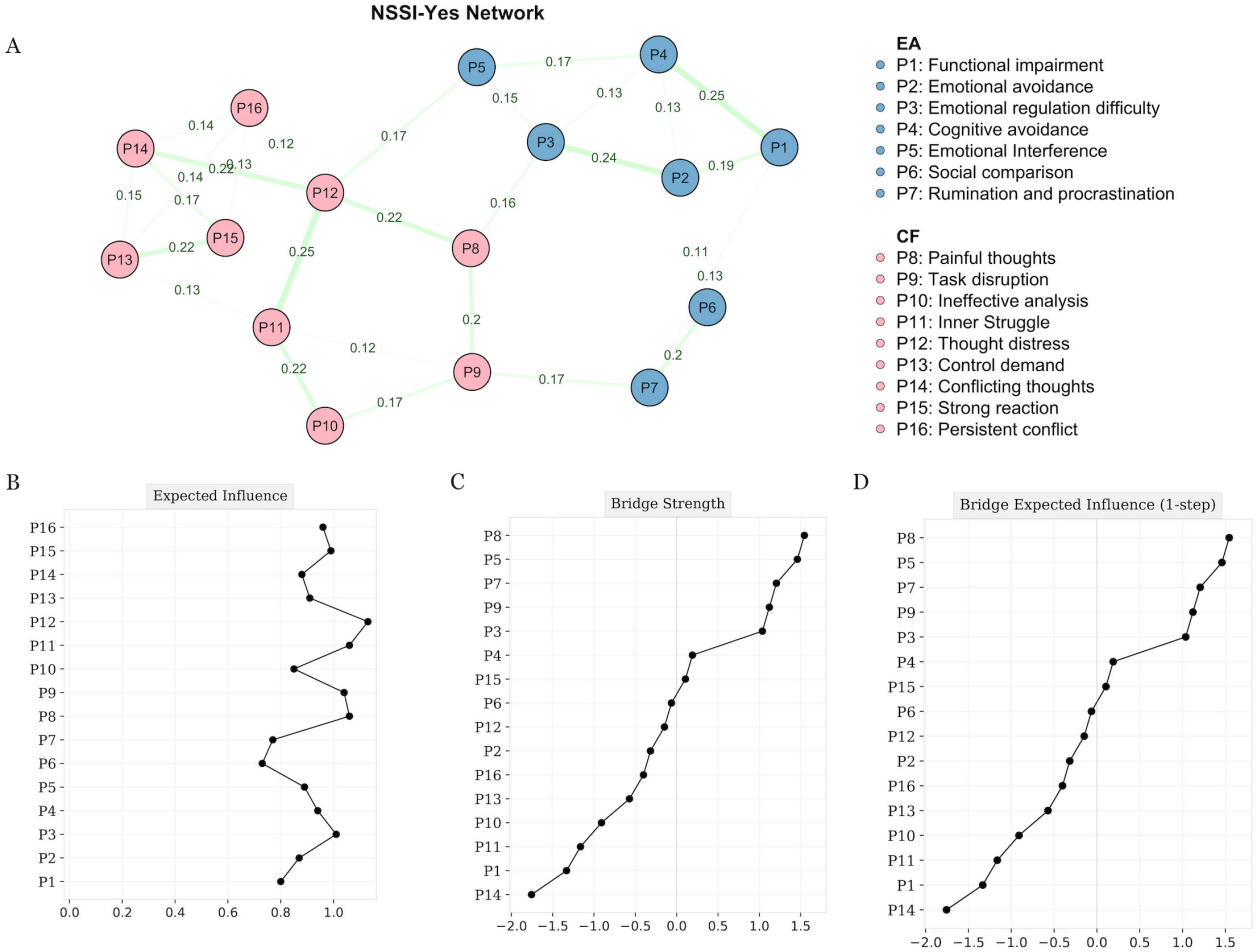
**(A)** Network Structure of Experiential Avoidance and Cognitive Fusion; **(B)** Expected Influence (EI) values in the network of experiential avoidance and cognitive fusion; **(C)** Bridge Strength values of bridging variables in the network of experiential avoidance and cognitive fusion; **(D)** Expected Influence (EI) values of bridging variables in the network of experiential avoidance and cognitive fusion.

This study employed network analysis to assess the relationships between EA and CF subscales in the NSSI subgroup (N = 1, 387). **Figure 1A** presents the results of the network structure analysis. The study found that the CF community exhibited strong internal connections within the network, particularly with P12 (“*I feel upset about certain thoughts*”) showing significant positive connections with P11 (“*I struggle with some of my thoughts*”, weight =0.25), P14 (“*Certain thoughts confuse me*”, weight=0.22), and P8 (“*Certain thoughts cause me distress and pain*”, weight=0.22).

Regarding the EI of the nodes, **Figure 1B** displays the centrality of all nodes. The centrality estimates indicate that P12 (“*I feel upset about certain thoughts*”) from CF had the highest EI value (EI = 1.13), suggesting that changes in this node significantly impact the entire network.

For the bridging variables in the network, **Figures 1C, D** present the bridging centrality indices, including bridge strength and BEI. Applying the 80^th^ percentile cutoff, four nodes were identified as key bridges between the EA and CF communities: P8 (“ Some thoughts make me feel distressed and troubled”, BEI = 0.44), P5 (“Emotions cause problems in my life”, BEI = 0.43), P7 (“Worries get in the way of my success”, BEI = 0.40), and P9 (“ I am so bothered by certain thoughts that I can’t accomplish what I need to do”, BEI = 0.39). Notably, these bridging nodes were distributed across both EA (P5, P7) and CF (P8, P9) communities, indicating that cross-community connections involve nodes from both domains rather than being concentrated in one. Full centrality and bridge centrality indices for all nodes are presented in **Supplementary Table 3**.

Finally, in terms of the stability of node centrality, the correlation stability coefficient for EI was 0.75, indicating excellent stability of this measure. **Supplementary Figures 1** and **S2** show the stability of EI centrality and the accuracy of edge weights, while **Supplementary Figures 3** and **S4** present the bootstrapped difference test results for node centrality and edge weights.

### 3.3 Sensitivity analysis

To assess whether the network structure was driven by comorbid depression and anxiety, we conducted a sensitivity analysis controlling for PHQ-9 and GAD-7 scores. The adjusted network showed high consistency with the original: P12 (thought distress) remained the most central node, and the same four bridging nodes (P8, P5, P7, P9) were identified at the 80^th^ percentile threshold. Rank-order correlations between the original and adjusted networks were high for both EI (Spearman r_s_ = 0.97) and BEI (r_s_ = 0.99). The overall edge weight correlation was r = 0.998. Detailed comparisons are presented in **Supplementary Table 4**.

### 3.4 Sex-based network differences

**Figure 2:**
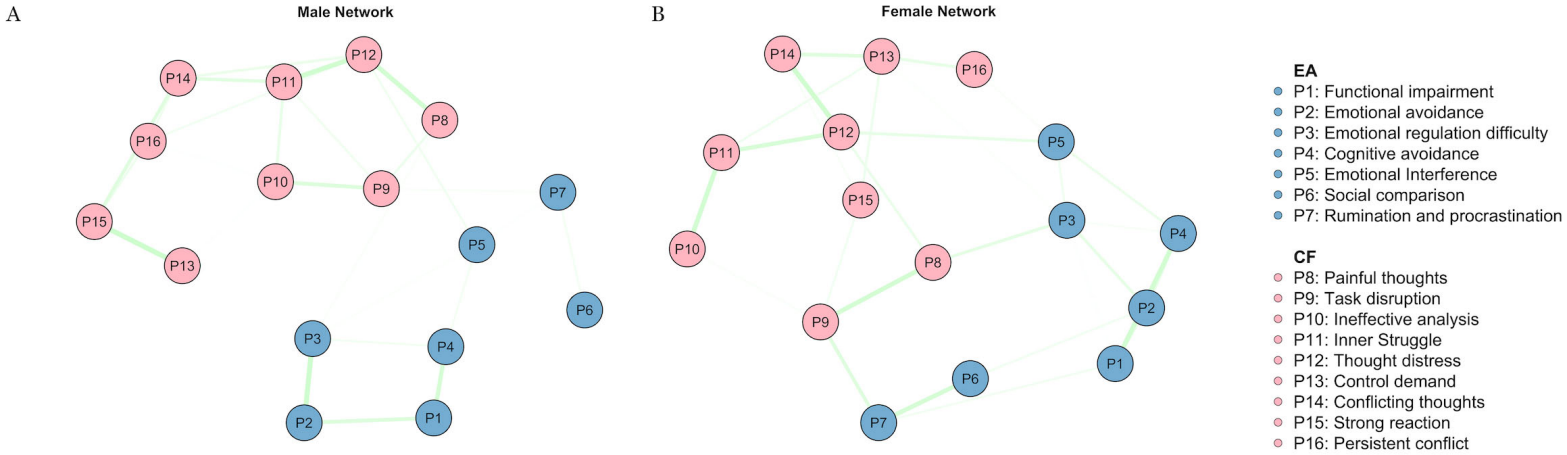
**(A)** Network structure of experiential avoidance and cognitive fusion in males; **(B)** Network structure of experiential avoidance and cognitive fusion in females. Nodes legends: Experiential Avoidance (Acceptance and Action Questionnaire – version II (AAQ-II)). P1—My painful experiences and memories make it difficult for me to live a life that I would value. P2—I’m afraid of my feelings. P3—I worry about being able to control my worries and feelings. P4—My painful memories prevent me from having a fulfilling life. P5—Emotions cause problems in my life. P6—It seems like most people are handling their lives better than I am. P7—Worries get in the way of my success. Cognitive Fusion (Cognitive Fusion Questionnaire). P8—Some thoughts make me feel distressed and troubled. P9—I am so bothered by certain thoughts that I can’t accomplish what I need to do. P10—I overanalyze certain situations, but it doesn’t help me at all. P11—I struggle with some of my own thoughts. P12—I feel upset about certain thoughts. P13—I need to control some thoughts that come to my mind. P14—Some thoughts leave me conflicted. P15—Some thoughts elicit strong reactions from me. P16—Although I understand it’s best to let go, I still get caught up in certain troubling thoughts. P1--Functional impairment; P2--Emotional avoidance; P3--Emotional regulation difficulty; P4--Cognitive avoidance; P5--Emotional Interference; P6--Social comparison; P7--Rumination and procrastination; P8--Painful thoughts; P9--Task disruption; P10--Ineffective analysis; P11--Inner struggle; P12--Thought distress; P13-- Control demand; P14--Conflicting thoughts; P15-- Strong reaction; P16--Persistent conflict.

The network structures of male and female adolescents with NSSI did not show significant differences in the NCT permutation test. Specifically, the network structure invariance test revealed no significant differences (maximum edge weight difference = 0.20, *p* = 0.07); the global strength invariance test also showed no significance (strength difference = 0.14, *p* = 0.46); and the edge weight variance test showed no significant differences for any of the edges (*p* > 0.05). The network structure of both groups is shown in **Figure 2**.

## 4 Discussion

To our knowledge, this is among the first studies to apply network analysis to explore the item-level interrelationships between EA and CF in adolescents. Through a psychometric network approach, we identified central and bridging symptoms that may underlie NSSI and inform potential targets for clinical intervention. The main findings included: 1) Thought distress (P12), a component of CF, was the most central node within the overall network; 2) Bridge analysis identified painful thoughts (P8), emotional interference (P5), rumination and procrastination (P7), and task disruption (P9) as key bridging nodes, distributed across both EA and CF communities; 3) There were no significant differences in overall network structure between male and female adolescents.

Our findings revealed that thought distress (P12) was the most central node, suggesting that interventions targeting distress associated with intrusive and maladaptive thoughts may have widespread effects across the cognitive-affective network, thereby alleviating a range of related symptoms. Within the NSSI population, individuals experiencing high levels of thought distress tend to report elevated anxiety and depressive symptoms, often leading to avoidance behaviors in an attempt to reduce distress. However, these short-term relief strategies may inadvertently reinforce CF, exacerbating psychological distress. Interestingly, prior research has found that individuals with high CF, but low EA do not necessarily exhibit significant psychological dysfunction (8), suggesting that EA may moderate the relationship between CF and mental health outcomes. This underscores the importance of promoting adaptive engagement with internal experiences in clinical settings to mitigate the negative consequences of CF.

Bridge analysis identified painful thoughts (P8), emotional interference (P5), rumination and procrastination (P7), and task disruption (P9) as key nodes connecting EA and CF symptom clusters. Notably, these bridging nodes were distributed across both communities—P5 and P7 from EA, and P8 and P9 from CF—indicating that cross-community connections involve nodes from both domains rather than being concentrated in one. P8 (painful thoughts) exhibited the highest BEI value, suggesting that distressing thought content may serve as a key conduit through which CF processes connect to EA responses. Meanwhile, P5 (emotional interference) highlights how disruption in daily functioning due to emotions links EA processes to CF. This pattern is consistent with the ACT framework, which posits that EA and CF are interrelated components of psychological inflexibility that tend to co-occur and interact to exacerbate distress (8, 31). The Experiential Avoidance Model (7) further suggests that NSSI functions as a negatively reinforced escape from unwanted internal experiences, and the bridging role of emotional interference (P5) and rumination (P7) is consistent with this model, as both reflect processes through which individuals struggle with, rather than accept, distressing experiences.

The sensitivity analysis confirmed that the EA-CF network structure was robust after controlling for comorbid depression (PHQ-9) and anxiety (GAD-7). The rank-order of both central and bridging nodes remained virtually unchanged, with Spearman correlations of 0.97 for EI and 0.99 for BEI between the original and adjusted networks. This indicates that the identified network properties are not merely artifacts of general psychological distress but reflect cognitive-affective patterns specific to the EA-CF relationship. These findings are consistent with the theoretical premise that EA and CF represent distinct yet interacting processes of psychological inflexibility (31), whose internal structure persists even when the variance attributable to common comorbidities is removed.

Despite significant sex differences in individual item responses, the NCT found no significant differences in overall network structure between male and female adolescents. One plausible explanation is the smaller proportion of males in the sample, which may have reduced the statistical power for detecting structural differences. Nonetheless, the generalizability of the network structure across sexes enhances the clinical relevance of our findings.

This study has several limitations, which we organize by design, measurement, and sampling considerations.

Regarding study design, first, the cross-sectional nature of the data precludes causal inference; longitudinal designs are needed to examine how changes in EA and CF influence the emergence and maintenance of NSSI over time. Second, although the present NSSI subgroup (n = 1, 387) is substantially larger than those in most prior studies in this area, the sample was sex-imbalanced, and NSSI group membership was defined by the presence rather than the severity of self-injurious behavior. Future work with sex-balanced samples and severity-stratified subgroups may reveal further nuances in network structure, particularly regarding sex-specific cognitive–affective pathways.

Regarding measurement, third, the psychometric adequacy of the AAQ-II for assessing EA has been questioned (32), and future studies would benefit from multidimensional instruments such as the Multidimensional Experiential Avoidance Questionnaire (MEAQ; Gámez et al. (33)). Fourth, NSSI behaviors were not modeled as network nodes; while this study focused on the internal structure of EA and CF as cognitive–affective processes relevant to NSSI, the absence of NSSI-specific nodes limits the ability to draw direct conclusions about how these network properties translate to self-injurious behavior. Incorporating NSSI frequency or severity as additional nodes in future research would allow a more direct examination of the pathways between cognitive–affective processes and self-injury outcomes.

Regarding sampling, fifth, participants self-reporting a history of psychiatric illness or prior suicidal behavior were excluded; because such self-report is subject to underreporting and because NSSI commonly co-occurs with psychiatric disorders and suicidality, generalizability to clinical populations is limited. Sixth, all participants were recruited from secondary vocational schools in China, which further limits generalizability to academic high-school students and to broader adolescent populations.

## 5 Conclusion

This study is among the first to apply network analysis to examine the fine-grained associations between EA and CF in adolescents with NSSI. The findings reveal that thought distress is a central node within the CF community, while painful thoughts, emotional interference, rumination, and task disruption serve as key bridging symptoms spanning both EA and CF communities and connecting two distinct yet interacting psychological processes. These symptom-level insights highlight potential intervention targets that may contribute to improving psychological flexibility. However, as NSSI behaviors were not modeled as network nodes, future research should directly incorporate NSSI symptoms into the network to validate these targets. Moreover, the stability of the network structure across sexes suggests the potential universality of these mechanisms. Future research should further investigate these dynamic relationships using longitudinal designs and integrate more comprehensive assessments of EA to optimize early detection and personalized intervention strategies in adolescents at risk for NSSI.

## Data Availability

The original contributions presented in the study are included in the article/**Supplementary Material**. Further inquiries can be directed to the corresponding author.
