# Supplementary material for: Cognitive-affective network structure in adolescents with non-suicidal self-injury: implications for clinical intervention
Source: Front Psychiatry. 2026 Jun 30;17:1804570. doi: 10.3389/fpsyt.2026.1804570 (PMC13366213; doi:10.3389/fpsyt.2026.1804570)
Supplement: Supplementary file 1 [file Supplementaryfile1.docx]

**Supplementary Materials**

**Table S1 Descriptive Statistics and Normality Indicators for Subscales of Experiential Avoidance(EA) and Cognitive Fusion(CF)**

**Table S2 Group Differences by Sex in EA and CF Subscales: Independent Sample t-test Results**

**Table S3 Centrality and Bridge Centrality Indices for All Nodes in the Network**

**Table S4 Sensitivity Analysis: Network Centrality and Bridge Indices Before and After Controlling for Depression (PHQ-9) and Anxiety (GAD-7)**

**Figure S1 Bootstrapped stability of the expected influence of the network analysis**

**Figure S2 Bootstrapped 95% confidence intervals for the edge weights of the network analysis**

**Figure S3 Bootstrapped difference test for node Strength centrality of the network analysis**

**Figure S4 Bootstrapped difference tests between edge-weights of the network analysis**

**Table S1 Descriptive Statistics and Normality Indicators for Subscales of Experiential Avoidance(EA) and Cognitive Fusion(CF)**

|  | Variables | M±SD | Skewness | Kurtosis |
| --- | --- | --- | --- | --- |
| Experiential Avoidance | P1 | 3.3±1.54 | 0.354 | -0.075 |
|  | P2 | 3.76±1.5 | 0.171 | -0.150 |
|  | P3 | 3.94±1.67 | 0.089 | -0.590 |
|  | P4 | 3.41±1.66 | 0.408 | -0.399 |
|  | P5 | 3.89±1.56 | 0.244 | -0.336 |
|  | P6 | 3.98±1.68 | 0.011 | -0.725 |
|  | P7 | 3.4±1.61 | 0.293 | -0.473 |
| Cognitive Fusion | P8 | 4.2±1.57 | 0.047 | -0.403 |
|  | P9 | 3.7±1.52 | 0.224 | -0.252 |
|  | P10 | 3.69±1.63 | 0.207 | -0.565 |
|  | P11 | 3.91±1.62 | 0.128 | -0.538 |
|  | P12 | 4.36±1.54 | -0.048 | -0.445 |
|  | P13 | 3.87±1.66 | 0.136 | -0.594 |
|  | P14 | 4.31±1.48 | -0.044 | -0.222 |
|  | P15 | 3.73±1.52 | 0.217 | -0.243 |
|  | P16 | 4.39±1.73 | -0.102 | -0.771 |

***Note.*** P1,Functional impairment;P2,Emotional avoidance;P3,Emotional regulation difficulty;P4,Cognitive avoidance;P5,Emotional Interference;P6,Social comparison;P7,Rumination and procrastination;P8,Painful thoughts;P9,Task disruption;P10,Ineffective analysis; P11,Inner struggle;P12,Thought distress; P13, Control demand;P14,Conflicting thoughts; P15, Strong reaction; P16, Persistent conflict

**Table S2 Group Differences by Sex in EA and CF Subscales: Independent Sample t-test Results**

|  | Variable | Male (N=667) | Female (N=720) | t | p |
| --- | --- | --- | --- | --- | --- |
|  | Age | 16.09 ± 0.94 | 15.93 ± 0.91 | 3.31 | 0.001 |
| Experiential avoidance | P1 | 3.17 ± 1.57 | 3.42 ± 1.5 | -3.10 | 0.002 |
|  | P2 | 3.42 ± 1.47 | 4.07 ± 1.46 | -8.30 | <0.001 |
|  | P3 | 3.57 ± 1.66 | 4.28 ± 1.6 | -8.11 | <0.001 |
|  | P4 | 3.13 ± 1.67 | 3.66 ± 1.62 | -5.96 | <0.001 |
|  | P5 | 3.54 ± 1.55 | 4.21 ± 1.5 | -8.28 | <0.001 |
|  | P6 | 3.75 ± 1.67 | 4.2 ± 1.66 | -5.02 | <0.001 |
|  | P7 | 3.21 ± 1.61 | 3.57 ± 1.6 | -4.13 | <0.001 |
| Cognitive Fusion | P8 | 3.88 ± 1.57 | 4.5 ± 1.5 | -7.53 | <0.001 |
|  | P9 | 3.45 ± 1.52 | 3.93 ± 1.48 | -5.93 | <0.001 |
|  | P10 | 3.44 ± 1.58 | 3.93 ± 1.64 | -5.67 | <0.001 |
|  | P11 | 3.58 ± 1.62 | 4.21 ± 1.57 | -7.45 | <0.001 |
|  | P12 | 4.03 ± 1.54 | 4.68 ± 1.48 | -8.06 | <0.001 |
|  | P13 | 3.62 ± 1.66 | 4.11 ± 1.62 | -5.58 | <0.001 |
|  | P14 | 4.04 ± 1.48 | 4.57 ± 1.43 | -6.85 | <0.001 |
|  | P15 | 3.54 ± 1.51 | 3.9 ± 1.51 | -4.42 | <0.001 |
|  | P16 | 4.07 ± 1.75 | 4.68 ± 1.66 | -6.61 | <0.001 |

***Note.*** P1,Functional impairment;P2,Emotional avoidance;P3,Emotional regulation difficulty;P4,Cognitive avoidance;P5,Emotional Interference;P6,Social comparison;P7,Rumination and procrastination;P8,Painful thoughts;P9,Task disruption;P10,Ineffective analysis; P11,Inner struggle;P12,Thought distress; P13, Control demand;P14,Conflicting thoughts; P15, Strong reaction; P16, Persistent conflict

**Table S3** Centrality and Bridge Centrality Indices for All Nodes in the Network

| **Node** | **Description** | **Domain** | **EI** | **Bridge Strength** | **BEI (1-step)** |
| --- | --- | --- | --- | --- | --- |
| P1 | Functional impairment | EA | 0.80 | 0.10 | 0.10 |
| P2 | Emotional avoidance | EA | 0.87 | 0.22 | 0.22 |
| P3 | Emotional regulation difficulty | EA | 1.01 | 0.38 | 0.38 |
| P4 | Cognitive avoidance | EA | 0.94 | 0.28 | 0.28 |
| P5 | Emotional Interference | EA | 0.89 | 0.43 | 0.43 |
| P6 | Social comparison | EA | 0.73 | 0.25 | 0.25 |
| P7 | Rumination and procrastination | EA | 0.77 | 0.40 | 0.40 |
| P8 | Painful thoughts | CF | 1.06 | 0.44 | 0.44 |
| P9 | Task disruption | CF | 1.04 | 0.39 | 0.39 |
| P10 | Ineffective analysis | CF | 0.85 | 0.15 | 0.15 |
| P11 | Inner Struggle | CF | 1.06 | 0.12 | 0.12 |
| P12 | Thought distress | CF | 1.13 | 0.24 | 0.24 |
| P13 | Control demand | CF | 0.91 | 0.19 | 0.19 |
| P14 | Conflicting thoughts | CF | 0.88 | 0.05 | 0.05 |
| P15 | Strong reaction | CF | 0.99 | 0.27 | 0.27 |
| P16 | Persistent conflict | CF | 0.96 | 0.21 | 0.21 |

*Note.* EI = Expected Influence; BEI = Bridge Expected Influence (1-step). Bridge centrality indices were computed from the EBIC-regularized partial correlation network (EBICglasso). EA = Experiential Avoidance (AAQ-II); CF = Cognitive Fusion (CFQ).

**Table S4** Sensitivity Analysis: Network Centrality and Bridge Indices Before and After Controlling for Depression (PHQ-9) and Anxiety (GAD-7)

| **Node** | **Description** | **Domain** | **EI (Original)** | **EI (Adjusted)** | **BEI (Original)** | **BEI (Adjusted)** |
| --- | --- | --- | --- | --- | --- | --- |
| P1 | Functional impairment | EA | 0.80 | 0.75 | 0.10 | 0.09 |
| P2 | Emotional avoidance | EA | 0.87 | 0.83 | 0.22 | 0.20 |
| P3 | Emotional regulation difficulty | EA | 1.01 | 0.91 | 0.38 | 0.33 |
| P4 | Cognitive avoidance | EA | 0.94 | 0.87 | 0.28 | 0.24 |
| P5 | Emotional Interference | EA | 0.89 | 0.78 | 0.43 | 0.36 |
| P6 | Social comparison | EA | 0.73 | 0.67 | 0.25 | 0.22 |
| P7 | Rumination and procrastination | EA | 0.77 | 0.77 | 0.40 | 0.40 |
| P8 | Painful thoughts | CF | 1.06 | 1.00 | 0.44 | 0.42 |
| P9 | Task disruption | CF | 1.04 | 0.98 | 0.39 | 0.36 |
| P10 | Ineffective analysis | CF | 0.85 | 0.81 | 0.15 | 0.14 |
| P11 | Inner Struggle | CF | 1.06 | 1.03 | 0.12 | 0.11 |
| P12 | Thought distress | CF | 1.13 | 1.05 | 0.24 | 0.21 |
| P13 | Control demand | CF | 0.91 | 0.85 | 0.19 | 0.17 |
| P14 | Conflicting thoughts | CF | 0.88 | 0.85 | 0.05 | 0.04 |
| P15 | Strong reaction | CF | 0.99 | 0.89 | 0.27 | 0.23 |
| P16 | Persistent conflict | CF | 0.96 | 0.85 | 0.21 | 0.16 |

**Stability Indices**

| **Metric** | **Value** |
| --- | --- |
| Most central node (Original) | P12 (Thought distress) |
| Most central node (Adjusted) | P12 (Thought distress) |
| Bridge nodes at 80th percentile (Original) | P5, P7, P8, P9 |
| Bridge nodes at 80th percentile (Adjusted) | P5, P7, P8, P9 |
| EI Spearman rank correlation | r_s = 0.97 |
| BEI Spearman rank correlation | r_s = 0.99 |
| Edge weight Pearson correlation | r = 0.998 |

***Note.*** Original = network estimated from raw item scores; Adjusted = network estimated from residuals after regressing out PHQ-9 (depression) and GAD-7 (anxiety) total scores from each item. EI = Expected Influence; BEI = Bridge Expected Influence (1-step). EA = Experiential Avoidance; CF = Cognitive Fusion. All network estimations used EBICglasso regularization.

**Figure S1**

**Bootstrapped stability of the expected influence of the network analysis**


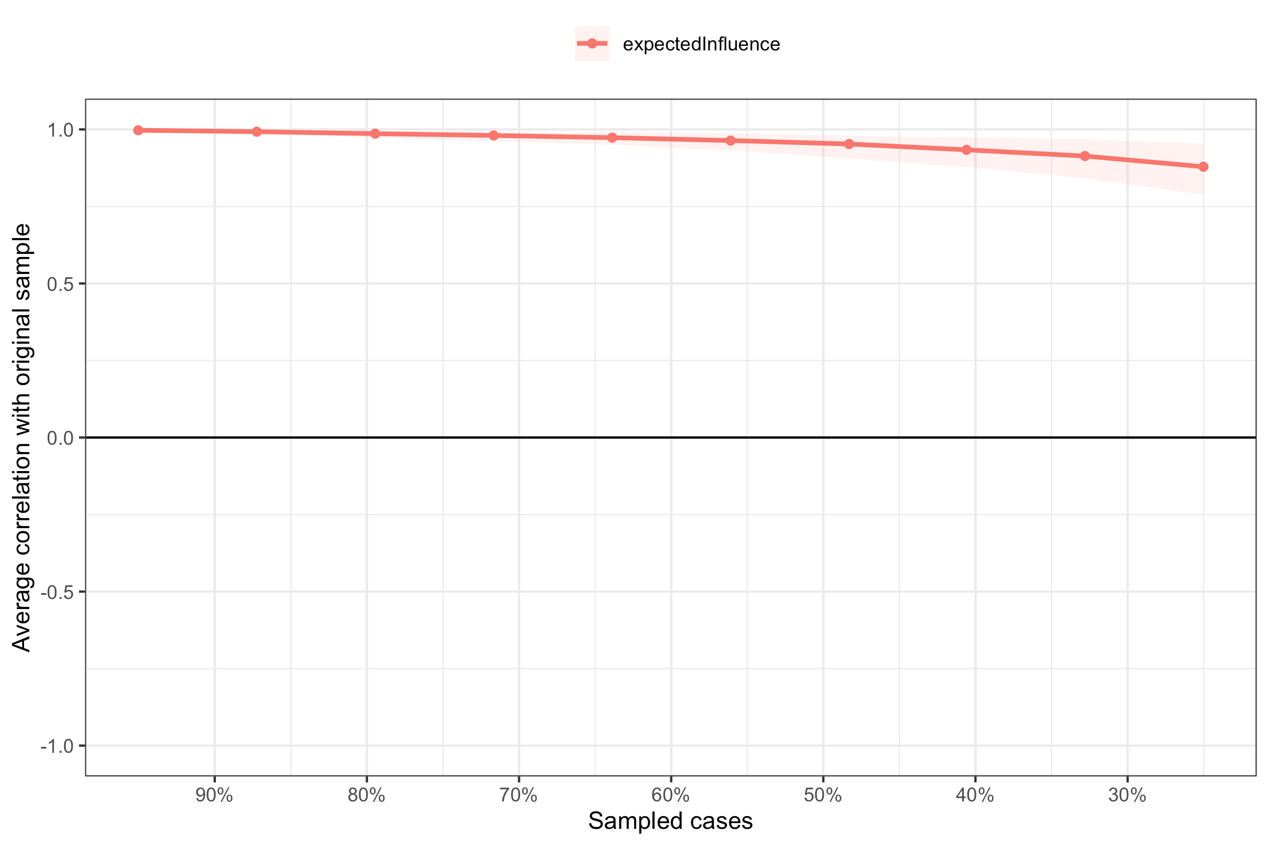


*Note: The Y-axis represents the average correlation coefficients, while the X-axis indicates the percentage of participants included in the subsamples. The stability of expected influence (a centrality measure) is assessed by repeatedly and randomly drawing subsamples from the original dataset and calculating the correlations between the expected influence in the subsamples and the original data. The correlation stability coefficient reflects the maximum proportion of participants that can be removed from the original sample while maintaining consistent results.*

**Figure S2**

*Bootstrapped 95% confidence intervals for the edge weights of the network analysis*
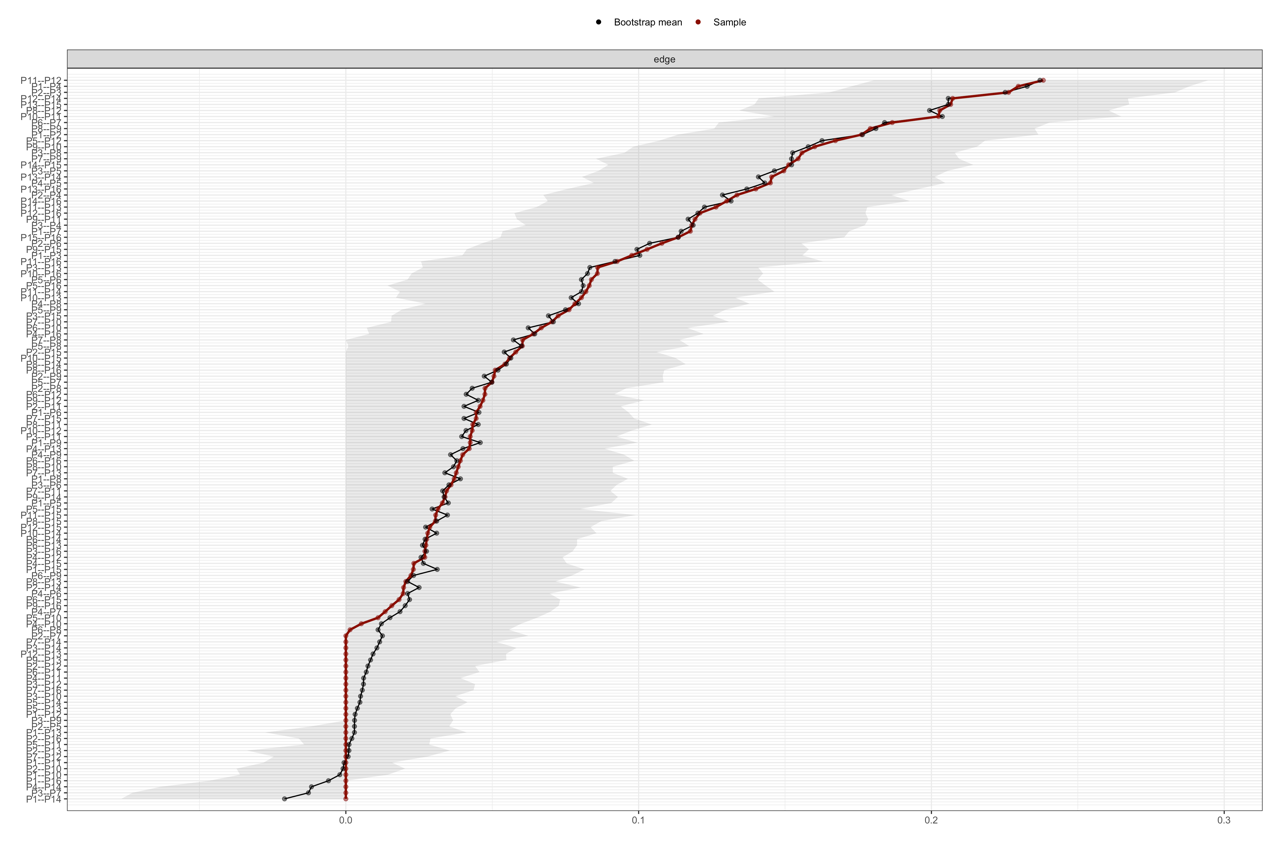


Note. Red dots represent the value (X-axis) of each edge weight from the network with NSSI-history; black dots, the value of each edge weight after passing the bootstrap stability test. Data are presented in the order of highest to lowest values. The gray area represents the 95% confidence interval of the edge weight; the wider the interval is, the lower the accuracy. Nodes represents the following: P1,Functional impairment;P2,Emotional avoidance;P3,Emotional regulation difficulty;P4,Cognitive avoidance;P5,Emotional Interference;P6,Social comparison;P7,Rumination and procrastination;P8,Painful thoughts;P9,Task disruption;P10,Ineffective analysis; P11,Inner struggle;P12,Thought distress; P13, Control demand;P14,Conflicting thoughts; P15, Strong reaction; P16, Persistent conflict

**Figure S3**

Bootstrapped difference test for node Strength centrality of the network analysis


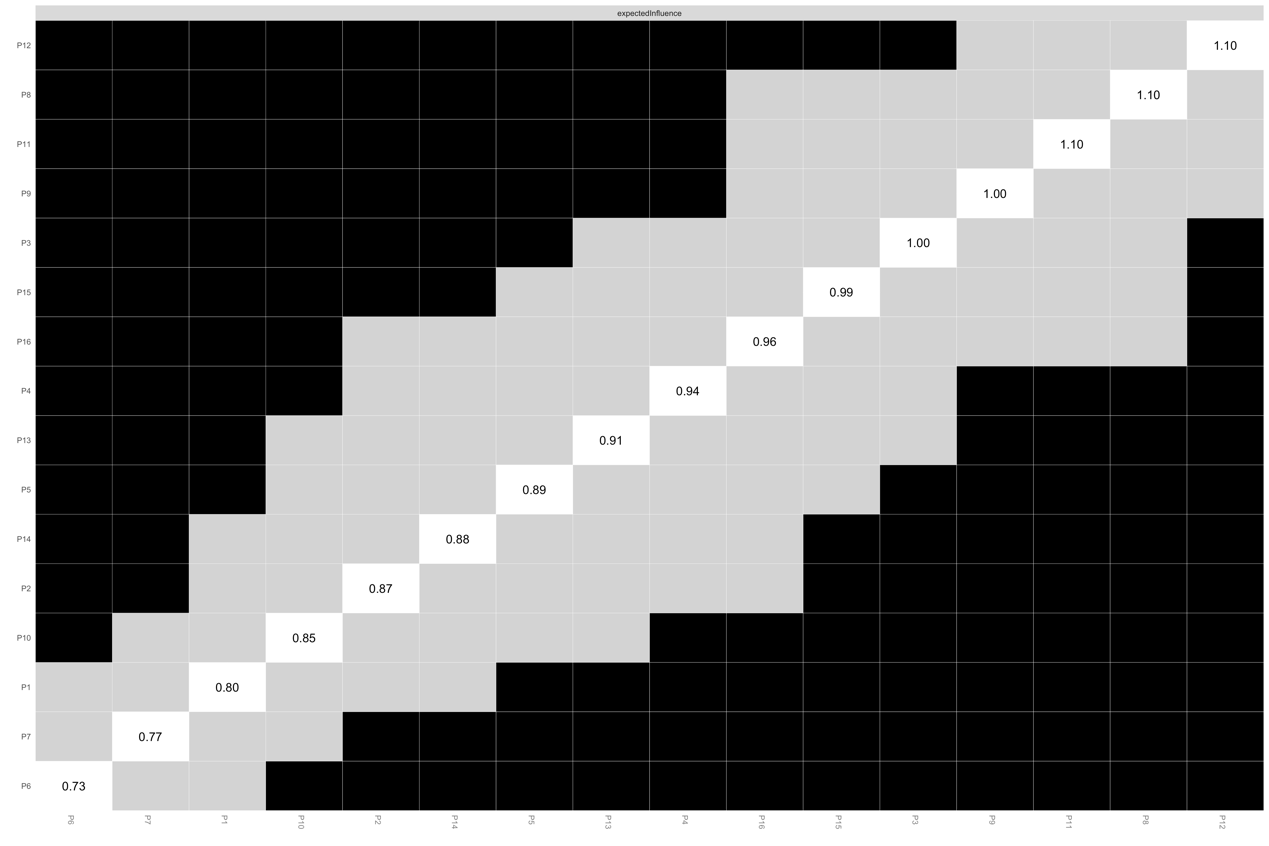


*Note.* Black boxes indicate Expected Influence (EI) that are significantly different from one another, and grey boxes indicate EI that are not significant different. Nodes represent the following: P1, Functional impairment; P2,Emotional avoidance;P3, Emotional regulation difficulty;P4,Cognitive avoidance;P5,Emotional Interference;P6,Social comparison;P7,Rumination and procrastination;P8,Painful thoughts;P9,Task disruption;P10,Ineffective analysis; P11,Inner struggle;P12,Thought distress; P13, Control demand;P14,Conflicting thoughts; P15, Strong reaction; P16, Persistent conflict.

**Figure S4** Bootstrapped difference tests between edge-weights of the network analysis


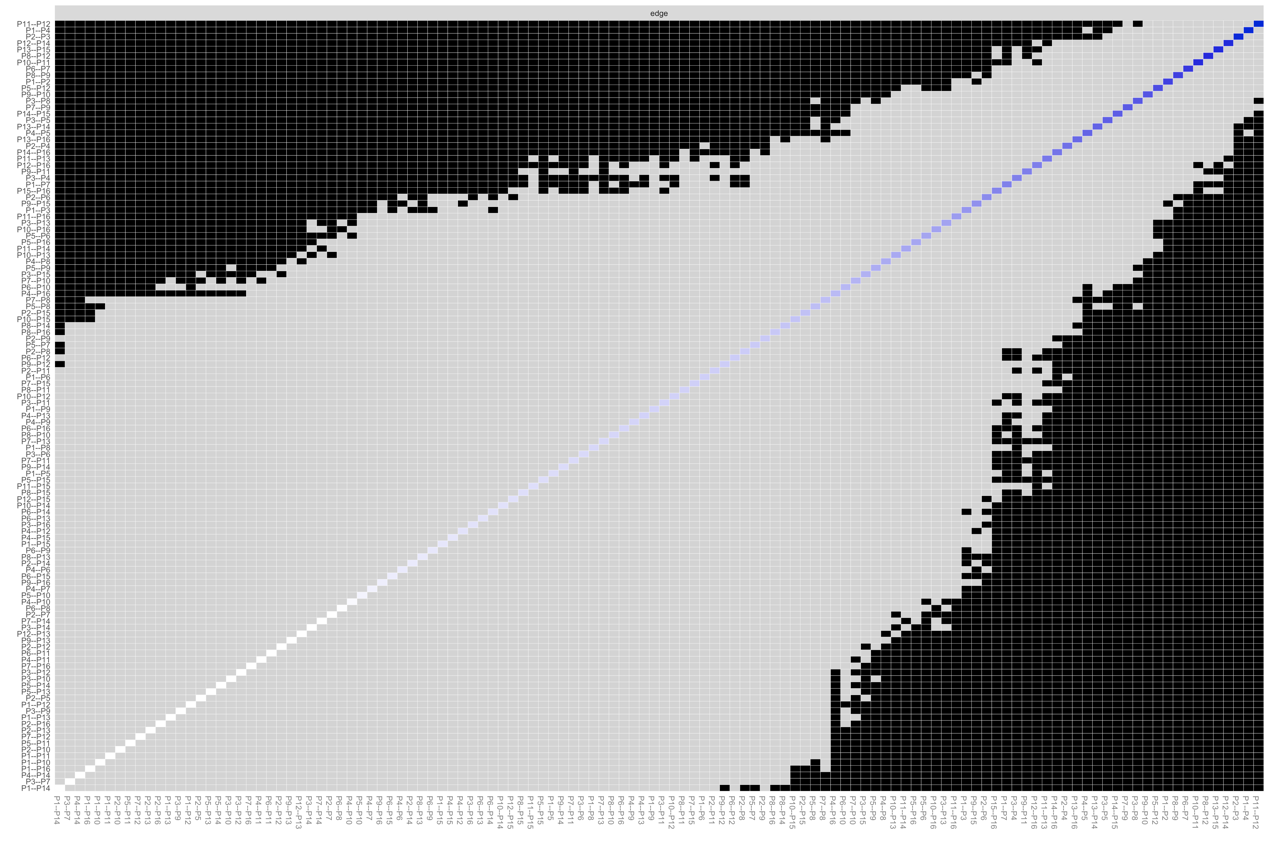


*Note.* Black boxes indicate Expected Influence (EI) that are significantly different from one another, and grey boxes indicate EI that are not significant different. Nodes represent the following: P1,Functional impairment; P2,Emotional avoidance;P3,Emotional regulation difficulty;P4,Cognitive avoidance;P5,Emotional Interference;P6,Social comparison;P7,Rumination and procrastination;P8,Painful thoughts;P9,Task disruption;P10,Ineffective analysis; P11,Inner struggle;P12,Thought distress; P13, Control demand;P14,Conflicting thoughts; P15, Strong reaction; P16, Persistent conflict.
